# Pathogen-derived HLA-E bound epitopes reveal broad primary anchor pocket tolerability and conformationally malleable peptide binding

**DOI:** 10.1038/s41467-018-05459-z

**Published:** 2018-08-07

**Authors:** Lucy C. Walters, Karl Harlos, Simon Brackenridge, Daniel Rozbesky, Jordan R. Barrett, Vitul Jain, Thomas S. Walter, Chris A. O’Callaghan, Persephone Borrow, Mireille Toebes, Scott G. Hansen, Jonah B Sacha, Shaheed Abdulhaqq, Justin M. Greene, Klaus Früh, Emily Marshall, Louis J. Picker, E. Yvonne Jones, Andrew J. McMichael, Geraldine M. Gillespie

**Affiliations:** 1 Nuffield Department of Medicine Research Building, Roosevelt Drive, Nuffield Department of Medicine, University of Oxford, Oxford, OX3 7FZ UK; 2 Division of Structural Biology, Wellcome Centre for Human Genetics, Roosevelt Drive, University of Oxford, Oxford, OX3 7BN UK; 3 Henry Wellcome Building for Molecular Physiology, University of Oxford, Oxford, OX3 7BN UK; 4 Department Molecular Oncology and Immunology, B6 Plesmanlaan 121, Amsterdam, 1066CX The Netherlands; 5 Vaccine and Gene Therapy Institute and Oregon National Primate Research Center, Oregon Health & Science University, Beaverton, OR 97006 USA

## Abstract

Through major histocompatibility complex class Ia leader sequence-derived (VL9) peptide binding and CD94/NKG2 receptor engagement, human leucocyte antigen E (HLA-E) reports cellular health to NK cells. Previous studies demonstrated a strong bias for VL9 binding by HLA-E, a preference subsequently supported by structural analyses. However, *Mycobacteria tuberculosis* (Mtb) infection and Rhesus cytomegalovirus-vectored SIV vaccinations revealed contexts where HLA-E and the rhesus homologue, Mamu-E, presented diverse pathogen-derived peptides to CD8^+^ T cells, respectively. Here we present crystal structures of HLA-E in complex with HIV and Mtb-derived peptides. We show that despite the presence of preferred primary anchor residues, HLA-E-bound peptides can adopt alternative conformations within the peptide binding groove. Furthermore, combined structural and mutagenesis analyses illustrate a greater tolerance for hydrophobic and polar residues in the primary pockets than previously appreciated. Finally, biochemical studies reveal HLA-E peptide binding and exchange characteristics with potential relevance to its alternative antigen presenting function in vivo.

## Abstract (web summary)

Human leucocyte antigen E (HLA-E) directly engages NK cells but also presents antigen to CD8^+^ T cells. Here the authors show crystal structures of HLA-E in complex with peptides derived from HIV and *Mycobacterium tuberculosis*, and describe binding conformations, the positional impact of residues involved and discuss implications for functional presentation to CD8^+^ T cells.

## Introduction

Human leucocyte antigen E (HLA-E) is a non-classical MHC class Ib molecule homologous to H-2 Qa-1 in mice and Mamu-E in rhesus macaques (RM)^1–4^. The two common human allotypes, HLA-E*01:01 and HLA-E*01:03, are essentially monomorphic, differing by a single amino acid substitution (Arg or Gly) at position 107, situated on a loop outside the peptide binding groove (PBG). Although relative expression is higher for HLA-E*01:03, both subtypes are present on the cell surface at lower levels than classical HLA-A or HLA-B molecules^3,4^. HLA-E exhibits preferential binding to a highly conserved set of nonameric signal (VL9) peptides derived from the leader sequence of HLA-A, B, C or G molecules^5–7^. Primary anchor residues are largely conserved among VL9 peptides: the canonical position 2 Met and position 9 Leu are accommodated by the primary B and F pockets, respectively. However, a position 2 Thr, present in a subset of HLA-B molecules, results in lower binding affinity to HLA-E and consequently, reduced surface expression^7–9^. VL9 peptide-bound HLA-E complexes engage the natural killer (NK) cell inhibitory receptor, CD94-NKG2A, thereby protecting healthy cells from NK cell-mediated lysis^6,9^. HLA-E also binds the NK cell activating receptor, CD94-NKG2C, although such interactions are of lower affinity^10^. Whilst VL9 peptide binding and NK cell regulation are ostensibly its primary function, HLA-E, and its rhesus and murine homologues, can present peptides from microbial and autologous sources to CD8^+^ T cells^3,11–21^. In *Mycobacterium tuberculosis* (Mtb) infection, multiple mycobacterial peptides have been shown to stimulate HLA-E restricted CD8^+^ T cells^15^. Similarly, Mamu-E restricted CD8^+^ T cell responses are elicited in RM by an experimental rhesus cytomegalovirus (RhCMV68-1) vaccine, recombinant for simian immunodeficiency virus (SIV) genes^13^. In both cases, the antigen presentation pathways are atypical, arising from Mtb-infected phagolysosomes in macrophage^22^ or vector-mediated disruption in RhCMV68-1-vaccinated macaques^13^. Remarkably, RhCMV68-1 stimulated SIV epitopes are particularly diverse with no simple sequence motif^13^, implying unexpectedly permissive peptide binding by Mamu-E^23^. These broad, Mamu-E restricted CD8^+^ T cell responses have been implicated as immune correlates of protection in RhCMV68-1 SIV vaccine studies^13^.

Here we explore pathogen-derived peptide binding to HLA-E from a structural and biochemical perspective. Through combined sandwich enzyme-linked immunosorbent assay (ELISA)-based and single-chain trimer approaches we confirm that the HLA-E peptide repertoire is broad. Via the mutagenesis and structural analysis of pathogen-derived peptides we also demonstrate an increased binding capacity of the primary pockets, with a greater breadth of tolerated anchor residues than originally reported for HLA-E-binding peptides^5,23–26^. This diversity also extends to the conformation of HLA-E-bound peptide which we show can differ dramatically from canonically orientated VL9, even in the presence of preferred primary anchor residues. Finally, we demonstrate that HLA-E is relatively stable without added peptide, favouring both low-affinity peptide binding and peptide exchange. These characteristics likely favour promiscuous peptide sampling in vivo, especially when the peptide loading complex (PLC) is disrupted, or absent in a peripheral intracellular compartment.

## Results

### Pathogen-derived peptide binding to HLA-E*01:03

**Fig. 1:**
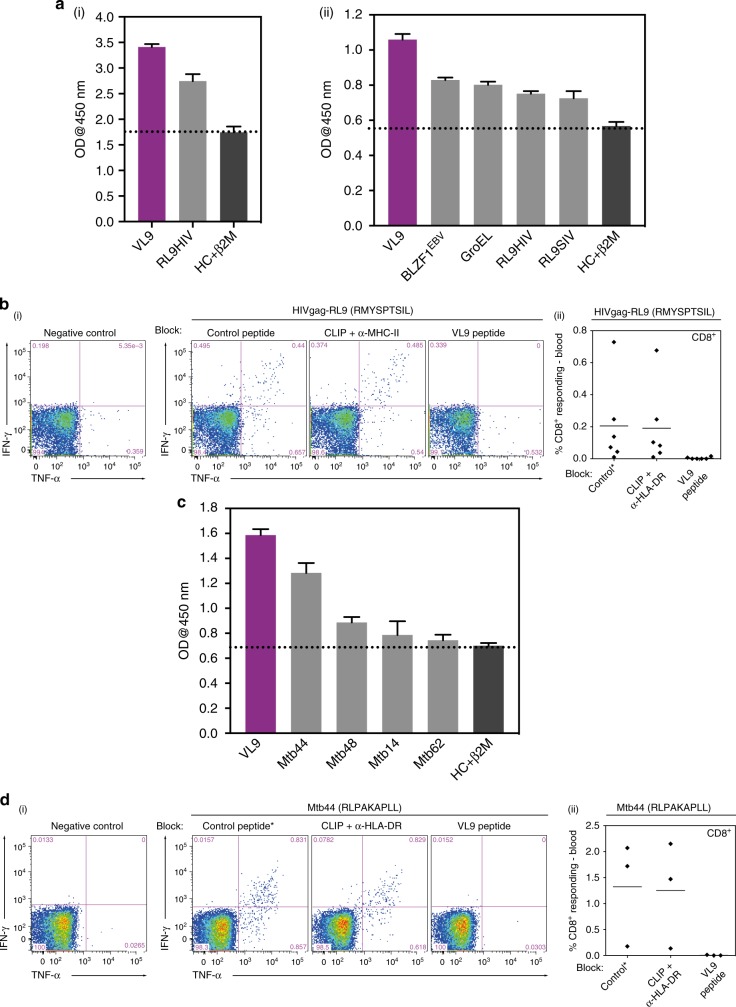
**a** Binding of RL9HIV and microbial peptides to human HLA-E. HLA-E (1 μM) and β2M (1.5 μM) were refolded with RL9HIV/RL9SIV peptide (70 μM) or with previously described HLA-E-restricted microbial epitopes. Following 48 h, HLA-E–β2M-peptide complex formation was assessed by sandwich ELISA. (**i**) Comparison of RL9HIV binding to VL9 (purple) and heavy chain (HC) + β2M only (dark grey) controls with peptide-binding cut-off noted (black dashed line). Data for five biological runs (two technical repeats/run) ± the standard error of the mean (SEM) is reported. (**ii**) Comparison of RL9HIV binding to HLA-E restricted microbial epitopes (one biological run and two technical replicas ± SEM). Colour coding as per (**i**). The *x* axes denote peptide identity and the *y* axes signify absorbance at 450 nm. **b** RhCMV68-1-HIV vaccinated rhesus macaques (RM) develop Mamu-E-restricted responses to the RL9HIV epitope. Peripheral blood mononuclear cells (PBMCs) from six RhCMV68-1-HIVGag vector vaccinated RM were stimulated with the RL9HIV peptide in the presence of either control peptide, CLIP peptide + anti-MHC-II mAb (MHC-II-blocking) or VL9 peptide (MHC-E-blocking). CD8^+^ T cell recognition was determined by evaluating interferon γ (IFNγ) and/or tumour necrosis factor (TNFα) production by flow cytometric ICS assays. Negative control reflects identical incubations but without added peptide. Representative flow cytometric profiles with response frequencies of gated CD3^+^, CD8^+^ T cells in each quadrant and frequencies of responding cells (IFNγ + and/or TNFα + ) in six vaccinated RM are reported. **c** Binding of *Mycobacterium tuberculosis* (Mtb)-derived peptides to human HLA-E. HLA-E (1 μM) and β2M (1.5 μM) were refolded with Mtb-derived peptide (70 μM). Following 48 h, HLA-E-β2M-peptide complex formation was quantified by sandwich ELISA. VL9 positive and HC + β2M only negative controls and the peptide-binding cut-off (black dashed line) are denoted. Data for three biological repeats (two technical replicas/repeat) with average absorbance ± SEM are reported. Plot axes as described in (**a**). **d** BCG-vaccinated RM develop Mamu-E-restricted responses to the Mtb44 epitope. Peripheral blood mononuclear cells (PBMCs) from three BCG-vaccinated RM were stimulated with the Mtb44 peptide in the presence of either control peptide, CLIP peptide + anti-MHC-II mAb (MHC-II-blocking) or VL9 peptide (MHC-E-blocking). Analysis of CD8^+^ T cell recognition as described in (**b**)

A micro-refolding and sandwich ELISA-based approach enabled relative quantification of peptide binding affinity for HLA-E*01:03. Two nonameric peptide panels were tested: the first included the HIVGag-derived, RMYSPTSIL (RL9HIV), a NetMHC predicted epitope^27^ homologous to the SIVGag-derived RMYNPTNIL peptide (RL9SIV), which constituted one of the two supertopes recognised by 100% of protected macaques in RhCMV68-1 vaccine trials^13^. Furthermore, since RL9HIV ranked as a strong binder relative to previously reported HLA-E restricted microbial peptides in the micro-refolding ELISA^25,28^ (Fig. 1a) and elicited Mamu-E restricted CD8^+^ T cell responses in RM vaccinated with an HIVGag-insert RhCMV68-1 vector (Fig. 1b), it was selected for crystallographic analysis.

A selection of published Mtb-derived peptides^15^ constituted the second panel, of which four facilitated HLA-E*01:03 complex formation in the micro-refolding ELISA (Fig. 1c). One peptide, RLPAKAPLL (Mtb44), exhibited comparable binding to that of the MHC class Ia-derived VL9 positive control peptide. As Mtb44 also elicited Mamu-E-restricted CD8^+^ T cell responses in Bacillus Calmette–Guérin (BCG) vaccinated RM (Fig. 1d), it was pursued in crystallographic studies.

### HLA-E*01:03—Mtb44 structure

**Fig. 2:**
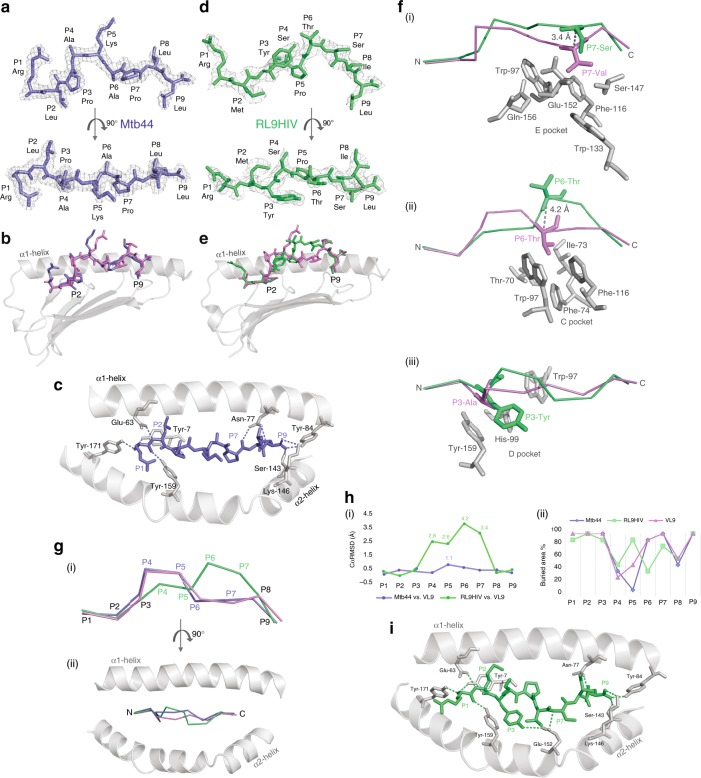
Structural analysis of HIV- and Mtb-derived peptide-bound HLA-E*01:03 complexes. **a** Mtb-derived Mtb44 peptide (RLPAKAPLL) visualised as sticks in purple-slate side-on and from above with electron density overlaid in grey mesh and HLA-E*01:03 HC + β2M omitted for clarity. (All electron density contoured at 1 sigma.) **b** Alignment of Mtb44 (purple-slate) and canonical VL9 (VMAPRTVLL) (violet) peptides depicted as sticks in the peptide binding groove displayed in grey cartoon with the omission of the α-2 helix for clarity. **c** Intermolecular Mtb44 peptide-HC hydrogen-bonding network visualisation. Peptide and HC-derived bonded residues displayed as solid sticks and H-bonds depicted as dashed lines (purple-slate). Peptide binding groove displayed as grey cartoon with the β-sheet floor omitted for clarity. **d** HIV-derived epitope “RL9HIV” (RMYSPTSIL) (lime green) in grey mesh electron density visualised side-on and from above with peptide binding groove omitted for clarity. **e** Alignment of RL9HIV (lime green) and canonical VL9 (violet) peptides depicted as sticks in the peptide binding groove with the α1 helix and β-sheet floor displayed in grey cartoon and the α2 helix omitted for clarity. **f** (**i**) E, (**ii**) C and (**iii**) D pocket visualisation for RL9HIV (lime green) superposed to VL9 (violet) with pocket-forming residues derived from the heavy chain of the RL9HIV-HLA-E complex depicted as grey sticks. Distances between the superposed peptide Cα atoms shown as grey dashed lines. **g** Cα backbone alignment of Mtb44, RL9HIV and VL9 peptides visualised side-on (**i**) and from above (**ii**) with peptide binding groove α1 + 2 helices depicted in grey cartoon. **h** (**i**) Distance in Å between superposed Cα atoms of Mtb44 versus VL9 and RL9HIV versus VL9 with peptide residue position along the *x* axis and distance on the *y* axis. (**ii**) Buried residue area percentage for Mtb44, RL9HIV and VL9 peptides with residue position along the *x *axis and buried area % on the *y *axis. **i** Intermolecular RL9HIV peptide-HC hydrogen-bonding network visualisation. Peptide and HC-derived bonded residues displayed as solid sticks with H-bonds depicted as dashed lines (lime green). Peptide binding groove displayed as grey cartoon with the β-sheet floor omitted for clarity

Previous crystal structures of HLA-E have been determined in complex with MHC class Ia leader sequence-derived VL9 peptides or HCMV UL40 protein-derived VL9 mimics^8,10,23^. Here we present the structure of HLA-E*01:03 bound to the Mtb-derived peptide, Mtb44 (RLPAKAPLL) (Fig. 2a). The complex packed in the P1 space group, diffracted to 2.1 Å and was assigned the PDB ID, 6GH1 (Supplementary Table 1). Clear electron density was visible in the PBG into which the Mtb44 peptide was modelled (Fig. 2a). Despite sequence disparity, superposition of Mtb44 and canonical VL9 revealed strong conformational similarity in peptide positioning (Fig. 2b). Both peptides adopt the classical kinked orientation in which the backbone arches away from the groove floor projecting residues four and five (P4&5) towards the solvent. Furthermore, eight of the nine canonical hydrogen (H) bonds connecting peptide and heavy chain (HC) are conserved with 1MHE. Formation of the Mtb44 P9-Leu [OXT] HLA-E Lys-146 [NZ] H-bond may counterbalance any loss of complex stability arising from the absent P5 H-bond, which connects VL9 to the α2 helix in 1MHE. Consistent with these structural observations and peptide binding assay data (Fig. 1 and Supplementary Table 2), Mtb44 complex stability is also underscored by thermal melt (Tm) analysis: Mtb44 shows a modest Tm increase (Tm = 50.2 °C ± 0.3) compared to the HLA-A2 leader sequence-derived VL9 peptide Tm documented here (Tm = 47.5 °C ± 0.4), and values previously reported for other MHC class Ia-derived leader sequence peptides^8,10^.

Analogous to previous VL9-bound structures, side chains of primary and secondary anchor residues in the Mtb44 complex project into their corresponding pockets towards the groove floor. Minimal repositioning of HLA-E-derived B pocket-lining residues suggests that P2 primary anchor Met to Leu substitution is well tolerated. Furthermore, the small side chains of P3-Pro and P6-Ala occupy the shallow D and C pockets, respectively.

### HLA-E*01:03—RL9HIV structure

Diffraction data from multiple isomorphous HLA-E*01:03—RL9HIV peptide crystals were merged in Xia2 yielding a 100% complete dataset to 2.6 Å resolution (Supplementary Table 1). The structure (PDB ID: 6GL1) was determined in the C2 space group with clear electron density visible in the PBG into which the RL9HIV peptide was modelled (Fig. 2d). RL9HIV positioning shows marked differences to the canonically orientated VL9 and Mtb44 peptides, in which P4 and 5 backbone arching ensures optimally positioned secondary anchor residues at P3, 6 and 7 for secondary D, C and E pocket binding, respectively (Fig. 2e). RL9HIV exhibits an alternative, C-terminally shifted, kinked motif, wherein P6 and P7 arch away from the base of the groove, disrupting C and E pocket occupancy. The P6 RL9HIV and VL9 Cα atoms are separated by 4.2 Å and the P7 Cα atoms by 3.4 Å (Fig. 2h (i)), disrupting the ability of the RL9HIV P6-Thr and P7-Ser side chains to occupy their respective C and E pockets (Fig. 2f (i) and (ii)), and concomitantly rendering this region of the peptide more solvent exposed (Fig. 2h (ii)). However, the extended conformation between P1 and 5 of the RL9HIV peptide, arising from C-terminally shifted backbone arching, positions the P5-Pro Cα atom only 1.8 Å from the VL9 P6-Thr Cα atom, with the two side chains deviating by as little as 1.2 Å, potentially permitting a degree of compensatory C pocket occupancy. Despite minimal Cα deviation facilitating optimal secondary anchor residue-pocket alignment, the P3-Tyr side chain is prohibitively large for shallow D pocket binding (Fig. 2f (iii)), instead projecting toward the α2 helix in the C-terminal direction of the peptide. This in turn triggers the D pocket lining residue, His-99, to adopt an alternative rotamer. An extensive, yet distinct, hydrogen-bonding network secures RL9HIV in the PBG (Fig. 2i). Six of the nine H-bonds connecting VL9 and HC in 1MHE are conserved in the RL9HIV complex. However, four novel bonds are formed, one of which (P9-Leu [OXT]—Lys-146 [NZ]) is also present in the Mtb44 complex. Glu-152, situated on the α2 helix, forms novel bonds with the P3-Tyr side chain and P7 backbone, the latter of which canonically bonds with Asn-77 on the α1 helix in 1MHE. Thus, P6 and 7 are stabilised closer to the α2 helix in RL9HIV than in other HLA-E-peptide complex structures.

### Analysis of Mtb44 primary anchor residue variants

**Fig. 3:**
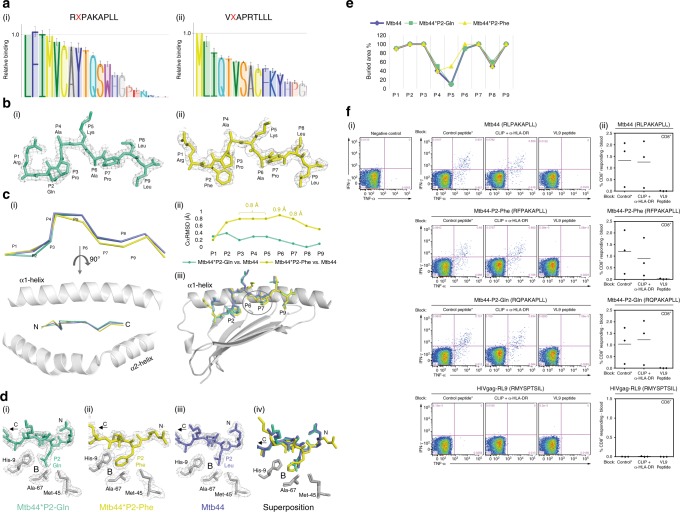
Structural analyses of Mtb44 position 2 peptide variants: Mtb44*P2-Gln and Mtb44*P2-Phe. **a** Relative binding of Mtb44 (**i**) and VL9 (**ii**) position 2 peptide variants from single-chain peptide-β2M-HC DNA constructs transfected in 293T cells and tested for HLA-E surface expression by flow cytometry using the MHC-E-specific 3D12 antibody. Data scaled relative to the index position 2 residue of each peptide (Leu for Mtb44 and Met for VL9). Relative binding displayed on the *y* axis, with position 2 residue mutations on the *x* axis. The mean ± SEM of the MFI is reported (*n* = 4). **b**: (**i**) The Mtb44 position 2 Glutamine variant Mtb44*P2-Gln (green-cyan) and (**ii**) the Mtb44 position 2 phenylalanine variant Mtb44*P2-Phe (yellow) visualised side-on with electron density overlaid in grey mesh and HC + β2M omitted for clarity. **c** (**i**) Cα backbone alignment of Mtb44*P2-Gln (green-cyan), Mtb44*P2-Phe (yellow) and Mtb44 (purple-slate) peptides visualised side-on and from above, respectively, with peptide binding groove α-1 + 2 helices depicted in grey cartoon. (**ii**) Distance in Å between superposed Cα atoms of Mtb44*P2-Gln versus Mtb44 and Mtb44*P2-Phe versus Mtb44 with peptide residue position along the *x* axis and distance (Å) on the *y* axis. (**iii**) Superposition of Mtb44*P2-Gln (green-cyan), Mtb44*P2-Phe (yellow) and Mtb44 (purple-slate) peptides with peptide binding groove α1 helix and β-sheet floor depicted in grey cartoon. **d** B pocket visualisation for (**i**) Mtb44*P2-Gln (**ii**) Mtb44*P2-Phe (**iii**) Mtb44 and (**iv**) Mtb44*P2-Gln/Mtb44*P2-Phe/Mtb44 superposition. HLA-E*01:03 HC-derived pocket-forming residues depicted as grey sticks with electron density overlay as grey mesh. **e** Buried residue area percentage for Mtb44*P2-Phe, Mtb44*P2-Gln and Mtb44 peptides with residue position along the *x* axis and buried area % on the *y* axis. **f** BCG-elicited Mamu-E-restricted Mtb44-specific CD8^+^ T cells recognise Mtb44 position 2 variants. Peripheral blood mononuclear cells (PBMCs) from 3 BCG-vaccinated RM were stimulated with the Mtb44 (RLPAKAPLL) peptide, the position 2 peptide variants [Mtb44*P2-Phe (RFPAKAPLL), Mtb44*P2-Gln (RQPAKAPLL)] and the HIVgag-derived RL9HIV peptide. CD8^+^ T cell recognition determined and shown as described in Fig. 1b

Peptides previously shown to bind HLA-E predominantly have Met at P2 for optimal B pocket binding, in addition to a strong F pocket binding preference for Leu at P9. We investigated these specificities using a single-chain peptide-β2m-HC construct, where P2 variants of the Mtb44 and VL9 peptides were tested for cell-surface expression in transfected 293T cells. As illustrated in Fig. 3a, the B pocket tolerated all hydrophobic side chains at P2, in addition to polar residues such as Gln, Ser and Thr. Despite Mtb44 and VL9 producing slightly varying hierarchies of binding for P2 substituted residues, cell surface expression was not supported by the charged residues Glu, Asp or Arg in either peptide. We then sought to analyse the ability of the B pocket to accommodate a selection of these hydrophobic and polar residues, some of which are present in HLA-E restricted microbial peptides, by crystal structure determination of Mtb44 P2 variant peptide complexes. Diffraction quality crystals could be grown for HLA-E*01:03 in complex with Mtb44 P2-Gln and Phe variants, termed Mtb44*P2-Gln (PDB ID: 6GH4) and Mtb44*P2-Phe (PDB ID: 6GGM), respectively. HLA-E*01:03 was also crystallised bound to an Mtb44 peptide in which the aliphatic hydrophobic P9 primary anchor, Leu, was substituted by the aromatic hydrophobic residue, Phe, termed Mtb44*P9-Phe (PDB ID: 6GHN).

Reflections from multiple isomorphous Mtb44*P2-Phe crystals were merged and integrated by Xia2, producing a dataset to 2.7 Å in the P1 space group. Similarly, Mtb44*P2-Gln crystallised in P1, however, diffraction data were collected to a higher resolution of 2.1 Å (Supplementary Table 1). Electron density was present for both peptides including the substituted P2 side chains (Fig. 3b). Superposition of Mtb44*P2-Gln and Mtb44*P2-Phe with the original Mtb44 complex structure revealed similarly orientated peptides in the PBG (Fig. 3c). Perhaps unsurprisingly, considering its depth and hydrophobicity, P2 Leu to Gln or Phe substitutions were tolerated by the B pocket with minimal repositioning of the pocket-forming residues (Fig. 3d).

However, the P2 Mtb44*P2-Phe Cα atom is elevated 0.7 Å away from the groove floor in the direction of the α1 helix, relative to Mtb44, permitting accommodation of the larger aromatic side chain (Fig. 3c). Additionally, P6 and 7 of the Mtb44P2-Phe variant sit 0.9 and 0.8 Å deeper in their respective C and E pockets, in turn reducing the height of the P5 kink and increasing the P5 buried area 40% relative to Mtb44 and Mtb44*P2-Gln (Fig. 3e). Furthermore, Mtb44*P2-Phe forms three additional H-bonds securing P1 and 2 in the groove. Such features align with single-chain trimer-based transfectant data demonstrating that Mtb44 P2-Phe drives the highest relative levels of HLA-E surface expression. However, these minor readjustments in peptide positioning due to P2 substitution do not disrupt immune recognition: CD8^+^ T cells isolated from the spleens of BCG-vaccinated RM mounted responses of similar magnitude when stimulated with Mtb44*P2-Phe, Mtb44*P2-Gln or the index Mtb44 epitope, emphasising the similarity in positioning of solvent exposed side chains and thus their antigenicity in vivo (Fig. 3f).

**Fig. 4:**
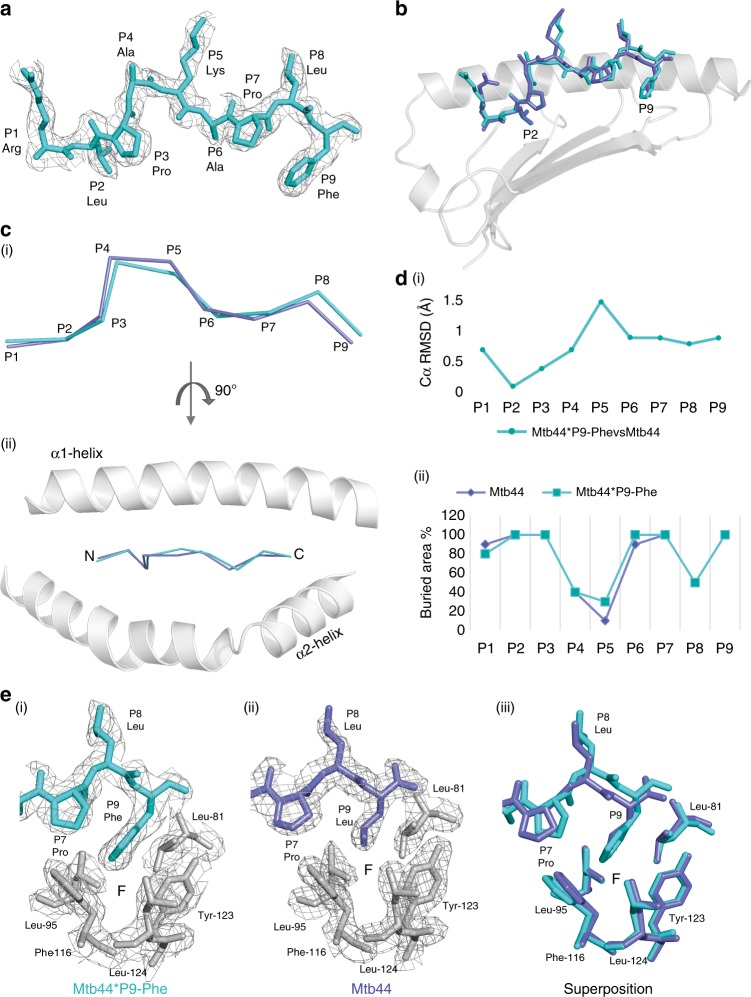
Structural analyses of the Mtb44 position 9 peptide variant: Mtb44*P9-Phe. **a** The Mtb44 position 9 phenylalanine variant Mtb44*P9-Phe (blue-cyan) visualised side-on with electron density overlaid in grey mesh and HLA-E*01:03 HC + β2M omitted for clarity. **b** Superposition of Mtb44*P9-Phe (blue-cyan) and Mtb44 (purple-slate) peptides depicted as sticks with the binding groove α1 helix and β-sheet floor depicted in grey cartoon. **c** Cα backbone alignment of Mtb44*P9-Phe (blue-cyan) and Mtb44 (purple-slate) peptides visualised side-on (**i**) and from above (**ii**), with binding groove α1 + 2 helices depicted as grey cartoon. **d** (**i**) Distance in Å between superposed Cα atoms of Mtb44*P9-Phe versus Mtb44 with peptide residue position on the *x* axis and distance (Å) on the *y* axis. (**ii**) Buried residue area percentage for Mtb44*P9-Phe (blue-cyan) and Mtb44 (purple-slate) peptides with residue position along the *x* axis and buried area % on the *y* axis. **e** F pocket visualisation of (**i**) Mtb44*P9-Phe (**ii**) Mtb44 and (**iii**) Mtb44*P9-Phe/Mtb44 superposed complexes. HLA-E*01:03 HC-derived F pocket-forming residues depicted as grey sticks with electron density overlaid as grey mesh

Reflections from multiple isomorphous Mtb44*P9-Phe complex crystals were merged in Xia2, yielding a dataset in the P1 space group to 2.5 Å. Clear electron density was present for the peptide including the substituted P9-Phe side chain (Fig. 4a). Similarly to the Mtb44 P2 variants, Mtb44*P9-Phe adopts the classical kinked conformation in the PBG and exhibits minor repositioning relative to the original Mtb44 peptide (Fig. 4b–d (i) and (ii)). Superposition revealed a slight elevation in the P9 Cα atom of Mtb44*P9-Phe compared to Mtb44 and an alternative rotamer for the F pocket-lining Phe-116 side chain, which tilts more acutely towards the groove floor, increasing pocket volume to accommodate the larger aromatic side chain (Fig. 4e). Minor repositioning also impacts the hydrogen-bonding network: three of the nine H-bonds securing Mtb44 to the groove are lost in Mtb44*P9-Phe at positions 1, 2 and 9.

### Blue-native (BN)-PAGE gel signatures of HLA-E complexes

**Fig. 5:**
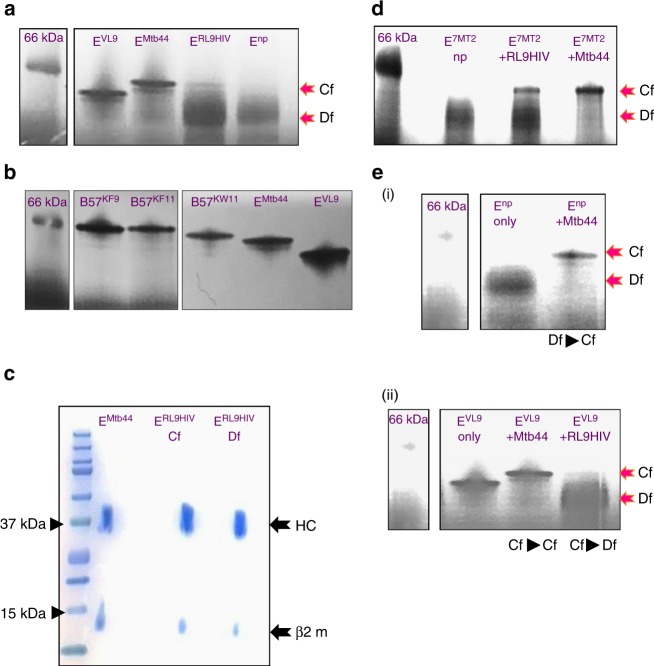
Blue-native-PAGE analysis of HLA-E complexes. **a** Blue-native (BN) gel signature of refolded HLA-E complexes. Purified HLA-E-β2m complexes refolded without peptide (no peptide (E^np^)) or in the presence of RL9HIV (E^RL9HIV^), Mtb44 (E^Mtb44^) or VL9 (E^VL9^) peptides were visualised by BN-PAGE gel. Red arrows indicate protein gel forms comprising compact (Cf) and diffuse (Df) species. The 66 kDa protein marker is highlighted for reference. **b** HLA-E compact (Cf) gel signatures resembled MHC class Ia folded proteins. Purified HLA-B57-β2m complexes refolded in the presence of the previously defined KAFSPEVIPMF (B57^KF11^), KAAFDLSFF (B57^KF9^) and KAYDTEVHNVW (B57^KW11^) epitopes were visualised by blue-native (BN) PAGE gel for comparison to HLA-E complexes refolded with VL9 and Mtb44. The 66 kDa protein marker is noted for reference. **c** 2D analysis of BN-gel resolved HLA-E-peptide refolds. BN-gel separated HLA-E-RL9HIV and HLA-E-Mtb44 complexes were 2D resolved via NuPAGE 10% Bis-Tris using standard 12-well lane gels where individual BN-gel slices were excised and individually separated. Heavy chain (HC) and β2m bands are noted (black arrows), and reference protein size markers are displayed (arrowheads). **d** BN-gel signatures of UV peptide labile-HLA-E complexes (E^7MT2^) following RL9HIV, Mtb44 and no peptide rescue. Pre-purified HLA-E molecules in complex with UV-labile 7MT2 were illuminated without peptide (np) or in the presence of RL9HIV and Mtb44 epitopes. The material was subsequently resolved by BN-gel. Red arrows indicate protein gel forms. The 66 kDa protein marker is noted for reference. **e** Exogenous peptide loading into no peptide and VL9-refolded HLA-E complexes. Purified HLA-E-β2m complexes refolded (**i**) without peptide (E^np^) were pulsed with 50 M excess Mtb44 peptide (**ii**) and pre-refolded HLA-E VL9 peptide complexes (E^VL9^) were pulsed with 200 M excess Mtb44 or RL9HIV peptides after which BN-gel signatures were analysed. Gel signature transitions are noted (text beneath images). Abbreviations throughout: *Cf* compact form; *Df* diffuse form

Despite the apparent homogenous nature of HLA-E refolded in the presence of RL9HIV, as indicated by size exclusion and ion-exchange chromatography, it was not possible to obtain a reproducible thermal melt pattern for this complex in contrast to VL9- and Mtb44-refolded HLA-E-β2m material. The immediate incorporation of dye suggested issues relating to sample nonuniformity and stability, and indicated that heterogeneous protein species—either higher order aggregates or mixed protein forms—were probably present. To explore this, we performed blue-native polyacrylamide gel electrophoresis (BN-PAGE) analysis to compare freshly purified RL9HIV-, Mtb44- and VL9-refolded HLA-E-β2m complexes. Unusually, HLA-E and β2M readily formed dimers in the absence of exogenously added peptide, and this material was also included in these experiments. The results (Fig. 5a) illustrate distinct gel signatures for the various HLA-E-β2m samples. For HLA-E refolded in the presence of the higher affinity binding Mtb44 and VL9 peptides, singular, compactly formed bands (“compact form” (Cf)) resolved downstream of the 66 kDa protein marker. The gel signatures of VL9- and Mtb44- refolded HLA-E complexes were similar in form, but positionally distinct, presumably due to the charge difference of solvent exposed peptide residues (two positively charged amino acids for Mtb44 and one for VL9). In contrast, HLA-E-RL9HIV complexes resolved heterogeneously as two main bands: a faint, Cf that resolved comparably to the HLA-E-Mtb44 Cf species, and a more dominant, diffuse form (Df). Finally, HLA-E-β2m refolded in the absence of peptide, resolved only as diffuse material similar to the Df observed for RL9HIV.

To determine if these gel signatures are comparable to classical MHC class I, the BN-PAGE gel profiles of HLA-B*57-β2m complexes refolded with epitope peptide were also explored. As depicted in Fig. 5b, Cf signatures exclusively represented all samples tested, confirming that the compact gel signature species most likely represents optimally folded, peptide-loaded MHC class I protein.

We next assessed whether the HLA-E Cf and Df species represented HLA-E-β2m co-complexes, as indicated by the presence of both the HC and β2m. This was particularly relevant for the Df species, as the native gel band size resolution error is high (~15%), and this smaller species could represent monomeric HC protein. To investigate this, Mtb44- and RL9HIV-refolded HLA-E-β2m complexes were probed in a second dimension by native/SDS-PAGE analysis. Individual Cf and Df bands resolved by BN-PAGE were individually excised and inserted in standard 12-well slots of NuPAGE 10% SDS-PAGE gels for subsequent separation. This approach verified the presence of HC and β2m in all folded forms (Fig. 5c).

We questioned whether pre-refolded HLA-E-β2m, with optimal tertiary structure and disulphide-bridge formation, was more receptive to RL9HIV peptide binding. To evaluate this, the method of UV-mediated peptide exchange was employed^29^. HLA-E refolded with the UV-labile VL9-based 7MT2 epitope incorporating a light sensitive J moiety at position 5 along the peptide was photo-illuminated in the presence or absence of 100 M excess Mtb44 and RL9HIV “rescue” peptides, and subsequently evaluated by BN-gel analysis. In accordance with observations made for conventionally refolded HLA-E complexes, Mtb44-exchanged material had a clear Cf signature whereas the RL9HIV fraction retrieved following peptide exchange comprised both diffuse and compact gel forms (Fig. 5d).

To assess whether HLA-E-β2m complexes, previously refolded in the absence of peptide, were peptide-receptive, their BN-gel signatures were evaluated following incubation with 50 M excess of Mtb44 peptide. As indicated by the transition of gel signatures from Df to Cf species (Fig. 5e(i)), peptide-receptivity was also a feature of the presumed peptide “empty” HLA-E-β2m forms.

The ability of HLA-E-β2m peptide-loaded material to exchange peptide was also evaluated. As Mtb44 and VL9-refolded HLA-E-β2m complexes produced BN-PAGE Cf gel signatures that are position-distinct, this feature was used as a tool to gauge peptide exchange. Previously refolded and purified HLA-E-β2m-VL9 peptide complexes were incubated with 200 M excess of Mtb44 peptide and subsequently analysed by BN-PAGE. As evidenced by the VL9 to Mtb44 Cf gel signature transition, VL9-loaded HLA-E complexes were readily displaced by the Mtb44 peptide (Fig. 5e(ii)). Finally, we tested whether the pre-refolded HLA-E-β2m-VL9 peptide complexes were also susceptible to RL9HIV peptide exchange. In agreement with observations made for the higher affinity Mtb44 peptide, 200 M excess of RL9HIV also displaced VL9 from HLA-E-β2m complexes, as evidenced by the emergence of gel forms resembling the HLA-E-β2m-RL9HIV pre-refolded complex signature (Fig. 5e(ii)).

## Discussion

Mamu-E restricted CD8^+^ T cell responses have been implicated as immune correlates of protection in RhCMV68-1-vectored SIV vaccination trials, triggering new interest in HLA-E as a potential driver of protective immunity against HIV-1^13,30–32^. Although earlier work indicated that the HLA-E binding repertoire was restricted to MHC class Ia leader sequence-derived peptides for presentation to NK cells, it is increasingly apparent that HLA-E can also bind and present autologous and microbially derived peptides to CD8^+^ T cells^3,15,18–20,33–36^. The breadth of epitopes identified in RhCMV68-1 SIV vaccine studies and mycobacterial infection^15^ indicates that both Mamu-E- and HLA-E-restricted peptide repertoires have the potential to be very diverse^13^. In particular, the range of Mamu-E restricted RhCMV68-1 stimulated responses is consistent with increased diversity of HLA-E peptide binding in transporter-associated with antigen processing (TAP)-deficient cell lines^37^. Despite sequence disparity between the human and rhesus homologues, the amino acids that comprise the five primary and secondary anchor residue-accommodating pockets are almost identical between HLA-E and 21 of the 22 Mamu-E alleles, with a single exception, I73T, in the C pocket (Supplementary Table 3). Furthermore, it has previously been shown that rhesus SIV-specific CD8^+^ T cells recognise peptides presented by both Mamu-E and HLA-E^13^. Similarly, rhesus CD8^+^ T cell responses to RL9HIV, Mtb44 or Mtb44 P2 variant epitopes were of comparable magnitude when peptides were presented by either Mamu-E or HLA-E (Supplementary Figs. 2, 3), further supporting the functional similarity between the rhesus and human homologues^38^. Finally, in a single-chain peptide-β2M-HC trimer-based comparison of VL9 P2 variant binding to HLA-E*01:03 versus Mamu-E*02:04, the human and rhesus homologues exhibited similar binding preferences (Supplementary Fig. 1A). Thus, although there are no published structures of Mamu-E, it is reasonable to extrapolate from these experimental data and the close relatedness of HLA-E that the two homologues behave similarly in folding and low-affinity peptide binding.

The breadth of the HLA-E peptidome was re-explored using a refolding-ELISA approach that quantified the relative capacity of peptides to stabilise HLA-E-β2M-peptide complex formation^8,39^ (Fig. 1a, c). Several HIV epitopes identified in RhCMV68-1 HIV-1 Gag-insert vaccine trials, including some that lacked canonical anchor residues, exhibited reproducible binding to HLA-E, albeit with considerably lower affinity than VL9 peptides (L. Picker, unpublished). Similarly, screens of previously reported HLA-E restricted microbial peptides, including an Mtb-derived panel, highlighted a selection that supported heterotrimeric complex formation including one, Mtb44, which exhibited comparable binding affinity to VL9 peptides. This assay also indicated that HLA-E and β2m-fold in the absence of added peptide, as evidenced by reproducible signals for peptide-free refolded samples.

As previous structures of HLA-E were crystallised in complex with highly conserved, canonical VL9 peptides or HCMV UL40 protein-derived VL9 mimics, it was unclear how nonleader sequence epitopes could be accommodated in the PBG. To understand the structural basis of binding, two of the highest affinity pathogen-derived peptides identified in refolding-ELISAs, RL9HIV (HIV gag) and Mtb44 (Mtb), were selected for crystallographic analysis (Figs. 1, 2). Irrespective of sequence disparity to VL9, including a positive charge at P1 and a primary anchor Met to Leu substitution at P2, Mtb44 exhibited strong conformational similarity, adopting the classical kinked binding motif in the PBG. However, despite a similar backbone conformation and solvent exposure profile to VL9, Mtb44 possesses sufficiently distinct exposed side chains to elicit specific, Mamu-E restricted CD8^+^ T cell responses in BCG-vaccinated macaques. In contrast, the RL9HIV peptide carries canonical anchors Met at P2 and Leu at P9, yet adopts an alternative C-terminally shifted kinked motif in the binding groove, resulting in a distinct solvent exposed signature with implications for immunogenicity and peptide-specific TCR interaction. This alternative backbone conformation, in turn, disrupts secondary C and E pocket occupancy with the P6 and 7 anchors projecting 4.2 and 3.4 Å further into the solvent, respectively, likely contributing to the lower overall stability of the complex.

In line with diverse and non-canonical SIVGag epitopes defined by Hansen et al.^13^, we provide structural evidence illustrating that the B and F pockets of HLA-E can tolerate a wider range of side chains than previously predicted (Figs. 3, 4). HLA-E structures crystallised in complex with Mtb44 variants encoding P2-Gln or Phe, or P9-Phe, demonstrated minimal conformational repositioning relative to Mtb44 in the PBG. However, these primary anchor mutations did have minor ramifications on complex stability, via the gain (for Phe at position 2) or loss (for the Phe position 9 variant) of three H-bonds. Notably, Mamu-E restricted Mtb44-specific CD8^+^ T cell responses in BCG-vaccinated macaques were preserved when the animals were challenged with mutant peptides harbouring Phe or Gln at P2. This supports the possibility of enhancing MHC-epitope complex stability via primary anchor optimisation of certain HLA-E-restricted epitopes in immunogen design without disrupting TCR recognition^40^. A more comprehensive analysis of B pocket tolerability was conducted by examining surface expression of single-chain peptide-β2M-HC trimers encoding Mtb44 P2 variants. The hierarchy of tolerated residues is consistent with our structural data, with P2-Phe up-regulating surface expression of HLA-E to the greatest degree. In fact, this assay indicates that the B pocket is capable of accommodating any hydrophobic residue, dramatically increasing the potential number of HLA-E restricted HIV-derived vaccine candidate epitopes. The single-chain-trimer data also indicate that tolerance of primary anchor substitution is to some extent dependent on the remaining amino acids that constitute the nonameric peptide, as when P2 substitutions in the Mtb44 and VL9 peptides were compared, HLA-E surface expression was up-regulated in different ranking orders.

During the biochemical characterisation of HLA-E, we noted that, unusually, HLA-E HC and β2m assembled as heterogeneous dimers in the absence of added peptide (Fig. 5). These dimeric forms likely comprised transition-state intermediates as evidenced by their diffuse gel profiles and the multiple transition states observed during thermal melt analysis. It is possible that peptide fragments derived from partially degraded HLA-E HC or β2M are present in the binding groove of these refolded species and it has previously been shown that peptide fragments as short as two amino acids (“dipeptides”) are capable of stabilising the F pocket^41^. However, it is not critical whether these refolded species are truly empty. More importantly, peptide-receptivity is a characteristic of these “empty” HLA-E-β2m forms, a finding that also concurs with data generated using particular peptide-free MHC class Ia-β2m heterodimers^42–44^. Intrinsic allotype-specific differences in the propensity of MHC folding intermediates to retain peptide-receptivity exist, presumably reflecting the extent to which different allelic forms maintain these stable functional states. In relation to PBG integrity in the absence of peptide, recent Molecular Dynamic Simulation studies predict that the α1–α2 helices of HLA-E are rigid and remain open when devoid of peptide, which contrasts with classical molecules such as HLA-A2 whose malleable helices are projected to collapse without peptide^13^. This, in addition to the apparent stability of the HLA-E-β2m peptide-free heterogeneous form identified here, may provide insight into the features that contribute to the broad peptide receptivity of HLA-E and Mamu-E where classical TAP-dependent peptide loading is blocked, for example, by various CMV genes and when RhCMV68-1 tropism is restricted to as yet undefined cell types in vivo^13^.

During our biochemical evaluation we also observed that HLA-E-bound VL9 leader peptide was readily displaced by exogenous challenge with Molar excess of the Mtb44 peptide. Additionally, an excess of lower affinity peptides appears to disrupt the integrity of VL9-refolded HLA-E complexes, as evidenced by the emergence of the “diffuse” dimer gel form upon challenge with the RL9HIV epitope. Remarkably, the highest affinity VL9 leader epitope imparts a relatively small increase (Tm ≤ 10 °C) to the stability of the HLA-E-β2m complex versus no peptide refolded forms, which contrasts the much larger values that high affinity peptides contribute to MHC class Ia stability upon binding^8,10^. Presumably this property of lower peptide affinity is driven from the perspective of NK cell recognition^45,46^. However, in the context-specific setting generated by the RhCMV68-1 regimen, this feature might enhance peptide-exchange properties of re-routed HLA-E-β2m complexes.

Based on the gel profiles of optimally loaded peptide complexes, it is highly likely that the homogeneous, Cf of HLA-E-RL9HIV represented material that crystallised in vitro. In contrast, the diffuse, peptide-receptive RL9HIV-specific material may comprise suboptimally bound peptide forms. The apparent incomplete loading of RL9HIV and other vaccine-identified peptides presumably reflects their lower affinities, given that exogenous loading of the higher affinity VL9 and Mtb44 peptides facilitated complete recovery of peptide-loaded, compact forms. It was initially reported that the MHC PBG undergoes conformational readjustments upon peptide binding^47^, with more recent hypotheses purporting that this is characterised by a two-stage transition where the groove initially exists in an “open” partially hydrated form accommodating suboptimally bound peptide, that converts ultimately, to a dehydrated, “closed” form upon optimal peptide loading^48,49^. A likely explanation is that the diffuse forms described here could include both “empty” heterodimer (open (o)) and weakly bound RL9HIV peptide (open-peptide (op)), of which the latter species transitions to the compact peptide bound form (Cp) at an equilibrium primarily influenced by the affinity of the epitope for HLA-E (Hβo ⇔ Hβop ⇔ Hβcp). Thus, for the higher affinity VL9 and Mtb44 peptides, the balance is strongly skewed to the compact peptide-bound forms, whereas for weaker epitopes such as RL9HIV, the equilibrium is shifted towards the open/open-peptide binding species. In Mtb and RhCMV68-1 infected cells we suggest that movement of peptide binding away from the quality control environment of the ER-based TAP-Tapasin-associated PLC to an alternative loading pathway potentially shifts the balance allowing low-affinity peptides with suboptimal sequence motifs (op forms) to bind HLA-E or Mamu-E. Whether MHC peptide editors such as TAP-binding protein related (TAPBPR) protein, further influence peptide selection in this alternative pathway is currently unknown^50–53^. The work of Hansen et al.^13^ suggests that RhCMV68-1 vaccinated macaques prime Mamu-E-restricted CD8^+^ T cells that subsequently recognise peptide targets on SIV infected cells following viral challenge^54^. The mechanism underlying how these low-affinity peptide epitopes elicit CD8^+^ T cell responses remains unclear. It is also unknown whether unusual forms of Mamu-E resembling the HLA-E ‘open’ material described here are generated in RhCMV68-1 vaccinated macaques, and whether CD8^+^ T cells recognise these forms. Both the mechanisms underlying alternative presentation routes and the features of peptide recognition by Mamu-E restricted CD8^+^ T cells are the focus of ongoing investigations.

## Methods

### Peptide synthesis

Synthetic nonameric peptides were generated by Fmoc (9-fluorenylmethoxy carbonyl) chemistry to a purity of 85% by Genscript USA. All peptides were provided as lyophilised power, reconstituted in DMSO to a concentration of 200 mM, and stored at −80 °C. A UV photolabile version of the HLA-B leader sequence peptide, VMAPRTLVL, incorporating a UV-sensitive 3-amino-3-(2-nitrophenyl)-propionic acid residue (J residue) substitution at position 5 (termed 7MT2), was synthesised by Dris Elatmioui at LUMC, The Netherlands. The 7MT2 peptide was stored as lyophilised power, and dispensed/reconstituted as required.

### RM and vaccines

A total of 9 purpose-bred male RM (Macaca mulatta) of Indian genetic background (3–7 years of age) were used in the animal experiments reported here. All animals were used with the approval of the Oregon National Primate Research Center (ONPRC) Institutional Animal Care and Use Committee, under the standards of the US National Institutes of Health Guide for the Care and Use of Laboratory Animals (IACUC). The ONPRC is accredited as a Category 1 facility by the American Association for Accreditation of Laboratory Animal Care (AAALAC) and has an approved Assurance (#A3304-01) for the care and use of animals on file with the Office for Protection from Research Risks at NIH. The IACUC adheres to national guidelines outlined in the Animal Welfare Act (7 U.S.C. Sections 2131–2159) and the Guide for the Care and Use of Laboratory Animals57 as mandated by the US Public Health Service Policy. The ONPRC IACUC approved care of RM, in addition to all experimental protocols and procedures. All RM were housed at the ONPRC in animal biosafety level (ABSL)-2 with autonomously controlled lighting, temperature and humidity. They were fed with commercially prepared primate chow (Purina Lab Diet: Fibre-Balanced Monkey Jumbo, 5000; High Protein Monkey Diet, 5045) twice daily and received daily supplemental fresh fruit or vegetables. Fresh, potable water was provided via automatic water systems. Physical exams, including body weight and complete blood counts, were performed at all protocol time points. A number of criteria were used to normalise the animals within vaccine groups, including MHC haplotype, age and sex. Once the groups were set, compatible animals within the same vaccine group were pair housed for the duration of the immunisation phase. Animals for which no compatible pair mate was identified, but otherwise met the pairing criteria, were single cage-housed for the duration of the immunisation phase or until a suitable compatible pair mate were identified. RM used in these experiments were free of cercopithicine herpesvirus 1, D-type simian retrovirus, and simian T-lymphotrophic virus type 1. Six RM (3–4 years of age) were subcutaneously vaccinated with the Rhesus cytomegalovirus 68-1 strain (RhCMV 68-1)^31^ expressing HIV-M-Gag-Nef fusion and HIV-M-Pol from the Episensus1 (RL9 RMYSPVSIL) or Episensus1 and Episensus2 (RL9 RMYSPTSIL)^55^. Three RM were immunised intravenously with 12.5 × 10^6^–1 × 10^8^ colony forming units (cfu) of TICE^®^ Strain Bacillus Calmette–Guérin (BCG) re-suspended in 3 mL of preservative-free saline.

### Generation of Mamu-E*02:04 transfectant

The Mamu-E*02:04 transfectant was generated as previously described via ligation of the allele into the pCEP4 plasmid, sequence confirmation of the gene insert, and then electroporation of the Mamu-E*02:04 pCEP4 plasmid using Nucleofector II/Kit C (Lonza) into the MHC-I null cell line K562^13,40^. Mamu-E transfectants were maintained on drug selection (Hygromycin B) and routinely confirmed for surface expression of MHC-I by staining with pan-MHC-I antibody clone W6/32. The HLA-E*01:03 transfectant was generously provided by Thorobald van Hall. In order to stabilise HLA-E and Mamu-E surface expression, transfectants were incubated at 27 °C for >3 h prior to use in assays and maintained at 27 °C throughout peptide incubation until combined with effectors used in assays and maintained at 27 °C throughout peptide incubation until combined with CD8^+^ effector cells. Surface MHC-E expression was confirmed via staining with W6/32 prior to use in T cell presentation assays.

### T cell assays

HIV- and Mtb-specific CD8^+^ T cell responses were measured in mononuclear cell preparations from blood by flow cytometric ICS, as previously described in detail^13,31^. Briefly, mononuclear cells were incubated with peptide and the costimulatory anti-CD28 (CD28.2: Purified; 500 ng/1e6 cells; eBioscience, 7014-0289-M050) and anti-CD49d mAbs (9F10: Purified; 500 ng/1e6 cells; eBioscience, 7014-0499-M050) for 1 h, followed by addition of 5 μg/mL Brefeldin A (BioLegend, 420601) for an additional 8 h. Costimulation without antigen served as a background control (no stim). MHC restriction of the response was determined by pre-incubating isolated mononuclear cells for 1 h at room temperature in the absence or presence of a control peptide (SIVgag-CM9 CTPYDINQM; 20 μM), anti-MHC-II block (anti-HLA-DR mAb; clone–G46.6; 10 μg/mL; BD Biosciences, 556642) and CLIP peptide block (MHC-II-associated invariant chain, amino acids 89–100; 20 μM), or the Mamu-E blocking peptide VL9 (VMAPRTLLL; 20 μM) prior to addition of test peptides. Stimulated cells were fixed, permeabilised and stained prior to flow cytometric analysis using an LSR-II instrument (BD Biosciences). Analysis was done using FlowJo software (Tree Star). In all analyses, progressive gating on the CD3^+^ population, and then the CD4^+^/CD8^−^ versus CD4^−^/CD8^+^ T cell subsets followed gating on the light scatter signature of small lymphocytes. Antigen specific CD8^+^ T cell response frequencies were determined from intracellular expression of IFN-γ and TNF-α. Boolean gates of (CD69^+^/TNF-α^+^ and/or CD69^+^/IFN-γ^+^) were determined on the gated (responding) CD8 + T cell population^56^. The following conjugated antibodies were used in these flow cytometric ICS analyses: anti-CD4 (L200: BV510; 10 ng/1e6 cells; BD Biosciences, 563094), anti-CD3 (SP34-2: Pacific Blue; 150 ng/1e6 cells; BD Biosciences, 558124), anti-CD8a (SK1: PerCP-eFluor 710; 7.5 ng/1e6 cells; eBioscience, 46-0087-42), anti-IFN-γ (B27: APC; 100 ng/1e6 cells; BD Biosciences, 624078), anti-TNF-α (MAB11: FITC; 180 ng/test; BD Biosciences, 624046 and PE; 180 ng/1e6 cells; BD Biosciences, 624049) and anti-CD69 (FN50: PE; 30 ng/1e6 cells; eBioscience, CUST01282 and PE/Dazzle 594; 60 ng/1e6 cells BioLegend, 310941).

### Protein production

HLA-E*01:03 HC residues 1–274 and β2-microglobulin (β2M) were cloned into PET22b prokaryotic vectors and expressed in BL21 competent *E.coli*. Inclusion bodies were purified via sonication and homogenisation in a Triton-based buffer prior to final re-solubilisation in 8 M urea, 50 mM MES pH 6.5, 0.1 mM EDTA and 0.1 mM DTT^23^.

### Peptide binding affinity assays

A peptide binding affinity assay was adapted from published micro-scale refold-ELISA-based methods^8,39^. In brief, 1 μM HC and 1.5 μM pre-refolded β2M were refolded in 0.33 mM Tris-Maleate and 0.5% Lutrol-F68 in the presence of 70 μM peptide, pre-diluted to 2 mM working stocks in 100 mM Tris-HCL pH 8. Micro-refolds were incubated at room temperature for 48 h before the relative capacity of each peptide to support stable HLA-E-β2M-peptide complex formation was quantified by sandwich ELISA. Correctly refolded heterotrimeric complexes diluted 1:100 in 2% IgG-free bovine serum albumin (BSA) were captured by the anti-human HLA-E monoclonal, 3D12 (10 μg/mL), in ELISA wells previously blocked and washed, respectively, with 2% BSA and 0.05% Tween-based wash buffer. 0.2 μg/mL polyclonal detection IgG raised in rabbits, specific for human β2M and enhancement antibodies specific for rabbit IgG, diluted 1:15 in 2% BSA, both conjugated to horseradish peroxidase, were sequentially added to ELISA wells to ensure detection of β2M-associated forms of HLA-E only. Tetramethyl benzidine substrate and STOP solution were used to develop and terminate reactions, respectively, before obtaining absorbance readings at 450 nm on a FLUOstar OMEGA plate reader.

### Protein refolding and purification

β2M (at a final concentration of 2 μM) was refolded in 100 mM Tris pH8.0, 400mM l-arginine monohydrochloride, 2 mM ethylenediamineteraacetic acid, 5 mM reduced glutathione and 0.5 mM oxidised Glutathione at 4 °C for 30 min before the addition of 20–50 μM peptide. HLA-E*01:03 HC was pulsed into the refolding buffer until a final concentration of 1 μM was reached. Following incubation for 72 h at 4 °C, HLA-E refolds were filtered through 1.0 μm cellular nitrate membranes to remove aggregates prior to concentration by centrifugation at 1000×*g* at 4 °C in Amicon Centricon Plus-70 and Ultra-15 10-kDa cut-off centrifugal filter devices. Samples were separated according to size into 20 mM Tris pH8, 100 mM NaCl by fast protein liquid chromatography on a Superdex S75 16/60 column. Elution profiles were visualised by UV absorbance at 280mAU, enabling differentiation of correctly refolded HLA-E-β2M-pepide complexes from smaller unassociated β2M and larger misfolded aggregates. Proteins were concentrated to 10 mg/mL for crystallisation and aliquots further analysed by SDS-PAGE electrophoresis to confirm presence of non-aggregated HLA-E HC and β2m.

### Crystallisation screening

A total of 100 nL protein, at 10 mg/mL, and 100 nL reservoir buffer were mixed in crystallisation wells and equilibrated by sitting drop vapour-diffusion at 20 °C^57^. Commercial sparse matrix grid screens were used to identify optimal crystallisation conditions, around which ammonium sulphate fine gradient and additive screens were subsequently setup (crystallisation buffer conditions for the five structures reported here are specified in Supplementary Table 1). Crystals were cryopreserved in 25% glycerol and diffraction data were collected at Diamond Light Source Beamlines I04 and I24. Data collection statistics are listed in Supplementary Table 1.

### Crystallographic analysis

Diffraction images from multiple isomorphous crystals were merged in Xia2 to increase completeness of the dataset^58,59^. Diffraction data were auto-indexed by Xia2 DIALS using the default parameters since 2015: I/sig(I) > 0.25, merged I/sig(I) > 1 and CC ½ > 0.5^58–60^. Initial phasing was carried out using the coordinates of the VL9-bound HLA-E*01:01 structure (PDB code 1MHE), stripped of peptide, hydrogens and waters as the search model in MolRep of the CCP4i suite^61–63^. Molecular replacement for RL9HIV and Mtb44 variant datasets was subsequently carried out in Phenix^64^ using the refined Mtb44 structure coordinates as the phasing model. Rigid body, restrained and TLS refinement were computed by CCP4i’s REFMAC5^61^ or Phenix.refine^65^ applying non-crystallographic symmetry restraints between iterative cycles of manual model building in Coot^66^. Models were validated using MolProbity^67^, visualised using the PyMOL Molecular Graphics System, version 2.0 (Schrödinger, LLC) and further investigated by PDBePISA^68^ and PDBeFOLD^69^.

### Design of peptide-β2m-HLA-E constructs

The coding sequence of mature HLA-E*01:03 HC, previously mutagenised to incorporate a position Y84A mutation, was PCR amplified using the forward primer (that also included a synonymous change creating a *Bam*H I restriction site), 5′-GACCTGGGCGGGaTCCCACTCCTTGAAGTATTTCC-3′, and the reverse primer 5-gtggatcCAAGCTGTGAGACTCAGACCC-3′^13,70^. This construct was inserted into pEGFP-N1 downstream of a *Hin*dIII–*Bam*H I cassette that contains the signal sequence of HLA-E*01:03, the coding sequence of the mature form of β2-microglobulin and a flexible [GGGGS]_4_ linker. Wild type and position 2 (p2) mutants of the VL9 and Mtb44 peptide sequences, followed by a flexible [GGGGS]_3_ linker, were introduced in between the HLA-E signal sequence and the start of the β2-microglobulin sequence by overlap extension PCR.

### Peptide-β2m-HLA-E transient transfection of 293 T cells

HEK 293T cells were maintained in 5% CO_2_ in DMEM (Life Technologies) supplemented with 10% Foetal Bovine Serum (SeraLabs), and Penicillin/Streptomycin (50 and 50 µg/mL, respectively, Life Technologies). Transfections were carried out at 70% confluency in six well plates using GeneJuice (Millipore) according to the manufacturer’s instructions. Following 24 h, cells were harvested. 1 million 293T cells were stained with 1 µL of the anti-HLA-E monoclonal antibody, 3D12 (BioLegend) in 100 µl PBS at 4 °C for 15 min. Cells were washed twice with PBS and stained with secondary antibody (allophycocyanin-crosslinked Goat-Anti-Mouse (H + L) F(ab’)2 fragment [Life Technologies]) diluted 1:1500 in PBS for a further 15 min. Subsequent to further (two) washing steps, cells were fixed in 100 µL of Cytofix (BD Biosciences), and acquired using a CyAn ADP Analyser (Beckman Coulter). Transfected cells were gated according to light scatter (Forward versus Side) and EGFP + HLA-E/Mamu-E co-expression (Supplementary Fig. 1). Data analysis were performed using FlowJo (TreeStar) software. Four biological repeats were included per construct.

### Thermal shift assay

The thermostability of refolded HLA-E-β2m complexes was determined by heat-induced fluorescent dye incorporation, using the commercially available Protein Thermal Shift Dye Kit^™^ (Applied Biosystems). In brief, 5 μg of test HLA-E-β2m complexes was aliquoted into 0.1 mL MicroAmp Fast Optical 96-well plates containing pre-mixed Protein Thermal Shift Dye and Protein Thermal Shift Buffer. Sample buffer (either PBS or Tris pH8, 100 mM NaCL) was added to achieve a final volume of 20 μL. Control samples reconstituted with buffer were prepared to monitor background fluorescent signal. Both samples and controls were set up in quadruplicate. Thermal-driven dye incorporation was measured on an Applied Biosystem Real-Time 7500 Fast PCR System. Data was collected over a temperature ramp ranging from 25 to 95 °C, with 1 °C intervals. Melt curve data were analysed using Protein thermal Shift Software v1.3, and median Derivative Tm values (°C) are reported.

### Blue-native polyacrylamide gel electrophoresis(BN-PAGE)

The composition of in vitro refolded HLA-E-β2m complexes was evaluated using the Blue Native-PAGE^™^ Novex Bis–Tris gel system (life technologies), in accordance with the manufacturer’s instructions (https://tools.thermofisher.com/content/sfs/manuals/nativepage_man.pdf). In brief, 3 μL of 4× Native-PAGE^™^ Sample Buffer was added to 10 μg (10 μL) of refolded HLA-E complexes, and immediately loaded on 3–12% Native-PAGE^™^ Novex Bis–Tris gels. NativeMark^™^ Unstained Protein Standard was used as the ladder control. Gel electrophoresis was performed at 150 Volts (with current gradient from 15–16 to 2–4 mAmps) for 2 h at room temperature. Following electrophoresis, gels were rinsed up to three times in MilliQ water prior to a 2–3 h staining step at room temperature in SimplyBlue™ SafeStain. De-staining was performed by multiple rounds of MilliQ water changes over a period of 24–48 h. Gel imaging was performed using a BioDoc IT Imaging System.

### 2D BN/SDS-PAGE analysis

2D SDS-PAGE separation was applied to samples previously resolved by Native-PAGE™ Novex Bis–Tris gel analysis. For the second dimension, SDS-PAGE NuPAGE 10% Bis–Tris gels of standard 12-well lane gels were used for individual protein band evaluation. To analyse the composition of distinct native gel species, individual bands were carefully excised using Smart Slicer plastic razor blades (LevGo Inc.), then reduced (in 1× NuPage LDS sample buffer, 50 mM DTT), alkylated (in 1× NuPage LDS with 50 mM DMA) and quenched (in 1× NuPage LDS supplemented with 20% ethanol and 5 mM DTT), prior to insertion into individual 1.5 mm wells of a 12-well NuPAGE gel. Electrophoresis was performed in MES buffer at a constant voltage of 200 for 35 min. Following electroporation, NuPAGE gels were rinsed three times in milliQ water prior to a 2 h staining step in SimplyBlue^™^ SafeStain. De-staining was performed in MilliQ water over a period of 24 h. Gel imaging was performed using a BioDoc IT Imaging System.

### Generation of HLA-E UV-labile monomers

Refolding of the VL9-based UV-labile 7MT2 peptide with HLA-E and β2M, and subsequent purification was performed as outlined previously^29^. For UV photo-cleavage and peptide exchange, 0.5 μM (~25 mg/mL) of UV-sensitive HLA-E-7MT2 monomer was incubated with 100 μM “exchange” peptide in polypropylene V-shaped 96-well plates (Greiner Bio-One), and the final volume was adjusted to 125 μL by adding exchange buffer (20 mM Tris, pH 7.4, 150 mM NaCl). UV exchange samples were incubated for 60 min on ice in a Camag UV cabinet with a long-wave 366 nm UV lamp. Subsequent to photo-illumination, samples were centrifuged at 4000×*g* to remove aggregated material. To obtain sufficient material for BN-PAGE gel analysis, UV exchange reactions were set up in quadruplicate, and following the removal of protein aggregates as described above, samples were pooled and concentrated by centrifugation at 13,000×*g* in Vivaspin 500 3 kDa MWCO micro-concentrators (Sartorius) to a volume of 20 μL prior to gel loading.

### Peptide exchange into pre-refolded HLA-E-β2M-VL9 complexes and no peptide refolded complexes

A total of 20 μg of purified HLA-E-β2m complexes, previously refolded without peptide or in the presence of VL9, were pulsed, respectively, with 50 or 200 M excess of Mtb44 or RL9HIV peptides for 2 h at room temperature, in a final volume of 20 μL. Then, 10 μL of each sample was loaded onto a BN-PAGE gel and their signature profiles were subsequently analysed.

### Data availability

Structural factors and atomic coordinates have been deposited within the Protein Data Bank with acquisition codes: 6GH1, 6GH4, 6GGM, 6GHN and 6GL1.

All relevant data outlined in this study are available from the authors.

## Electronic supplementary material

Supplementary Information
