# Author Correction: Pathogen-derived HLA-E bound epitopes reveal broad primary anchor pocket tolerability and conformationally malleable peptide binding

**DOI:** 10.1038/s41467-018-07304-9

**Published:** 2018-11-13

**Authors:** Lucy C. Walters, Karl Harlos, Simon Brackenridge, Daniel Rozbesky, Jordan R. Barrett, Vitul Jain, Thomas S. Walter, Chris A. O’Callaghan, Persephone Borrow, Mireille Toebes, Scott G. Hansen, Jonah B. Sacha, Shaheed Abdulhaqq, Justin M. Greene, Klaus Früh, Emily Marshall, Louis J. Picker, E. Yvonne Jones, Andrew J. McMichael, Geraldine M. Gillespie

**Affiliations:** 1 Nuffield Department of Medicine Research Building, Roosevelt Drive, Nuffield Department of Medicine, University of Oxford, Oxford, OX3 7FZ UK; 2 Division of Structural Biology, Wellcome Centre for Human Genetics, Roosevelt Drive, University of Oxford, Oxford, OX3 7BN UK; 3 Henry Wellcome Building for Molecular Physiology, University of Oxford, Oxford, OX3 7BN UK; 4 Department Molecular Oncology and Immunology, B6 Plesmanlaan 121, Amsterdam, 1066CX The Netherlands; 5 Vaccine and Gene Therapy Institute and Oregon National Primate Research Center, Oregon Health & Science University, Beaverton, OR 97006 USA

Correction to: *Nature Communications*; 10.1038/s41467-018-05459-z, published online 4 July 2018

The original version of this Article contained an error in the spelling of the author Jonah B Sacha, which was incorrectly given as Jonah Sacha. These errors have now been corrected in both the PDF and HTML versions of the Article.

