# Supplementary material for: Pathogen-derived HLA-E bound epitopes reveal broad primary anchor pocket tolerability and conformationally malleable peptide binding
Source: Nat Commun. 2018 Aug 7;9:3137. doi: 10.1038/s41467-018-05459-z (PMC6081459; doi:10.1038/s41467-018-05459-z)
Supplement: Supplementary file 1 — Supplementary Information [file 41467_2018_5459_MOESM1_ESM.pdf]

Supplementary Information

Pathogen-derived HLA-E bound epitopes reveal broad primary anchor pocket tolerability and conformationally malleable peptide binding

Walters et al.

Supplementary Figure 1:

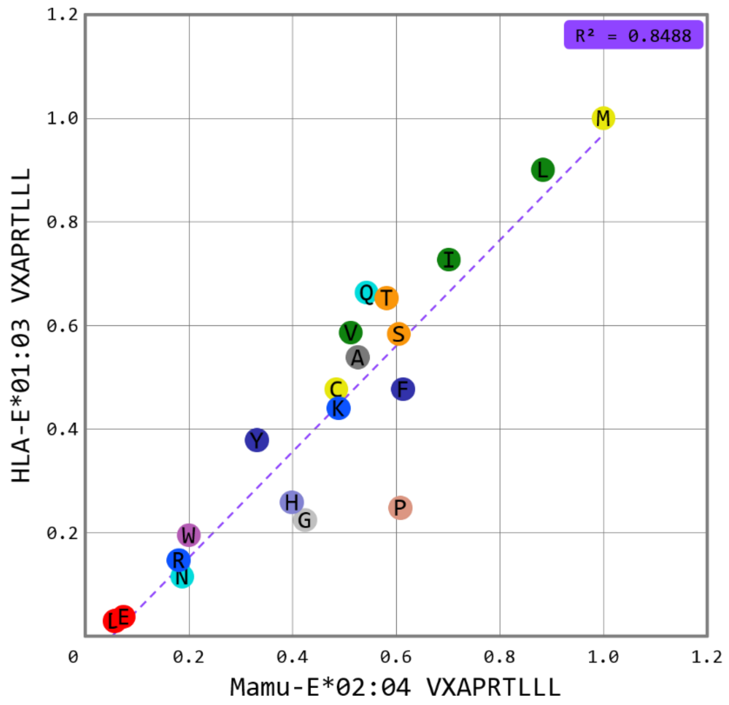

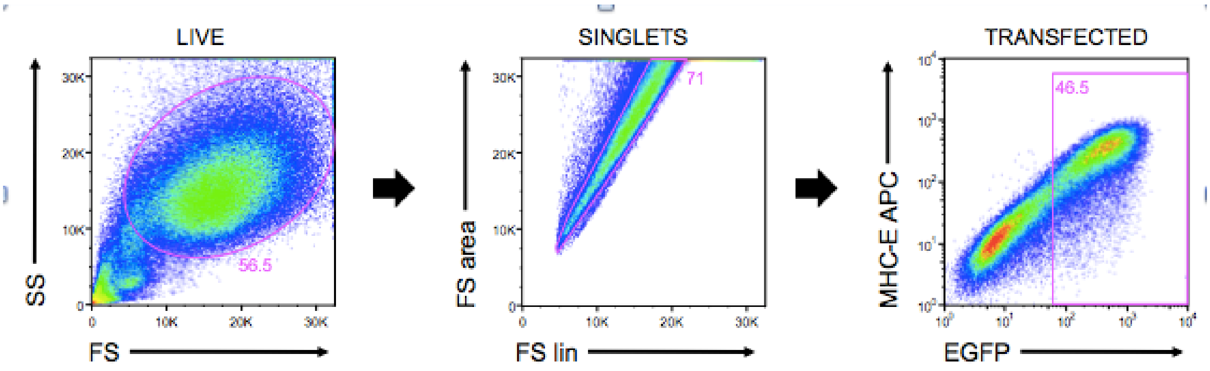

Comparison of HLA-E and Mamu-E single chain trimer surface expression

HLA-E (y axis) and Mamu-E (x axis) single chain (peptide-β2m-hc) constructs with VL9 peptides containing different amino acids at position 2 were transfected in 293T cells, and tested for HLA-E surface expression by flow cytometry using the MHC-E-specific 3D12 antibody. Transfected cells were gated according to light scatter (Forward versus Side) and EGFP expression. Data analysis was performed using FlowJo (TreeStar) software. Datasets were first corrected by subtraction of non HLA-E/Mamu-E binding peptides and normalized to the wild type VL9 peptide. N=4.

Supplementary Figure 2:

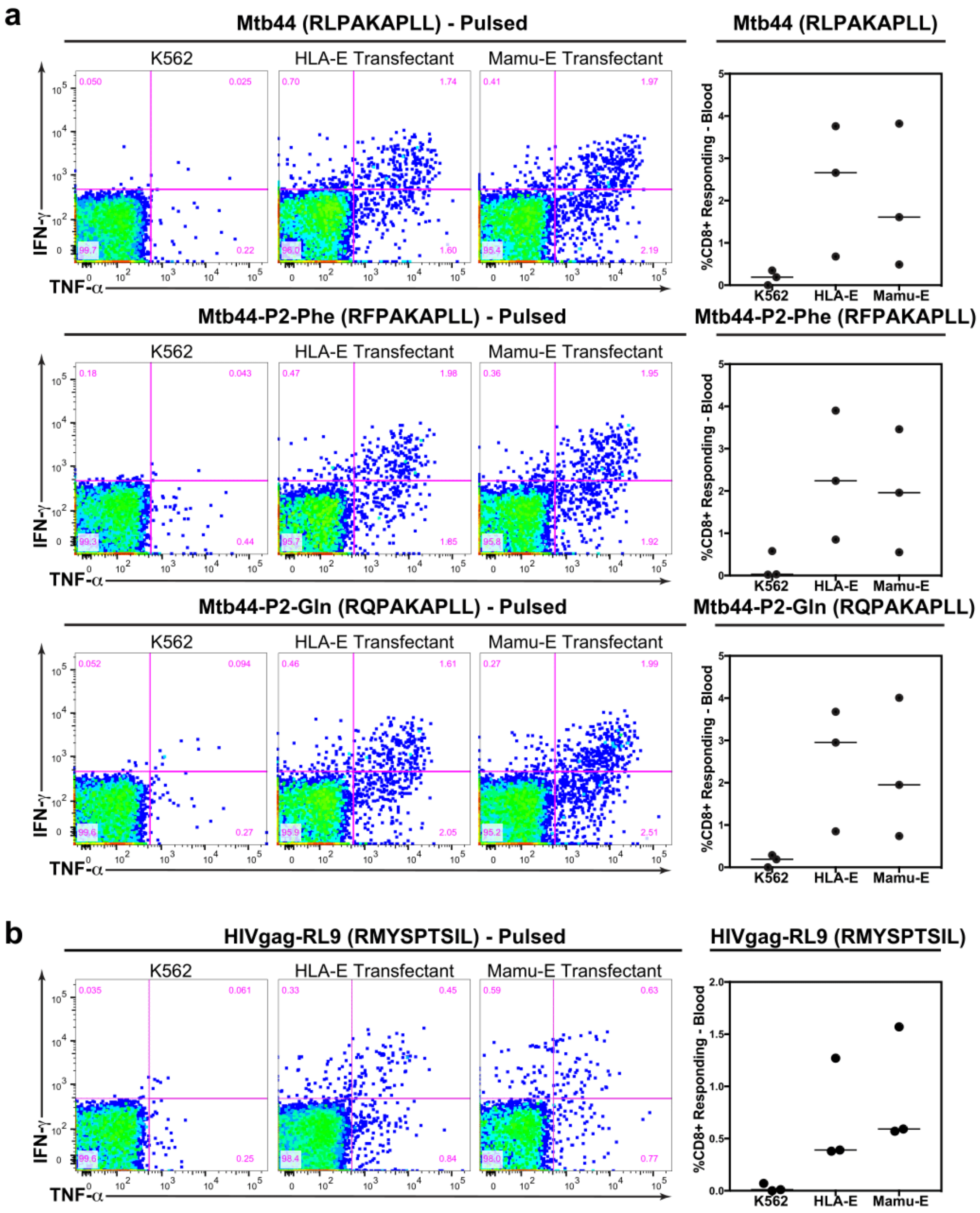

HLA-E and Mamu-E bind and present Mtb RL9 and HIV RL9 peptides equivalently to Mamu-E-restricted CD8+ T cells

MHC-I null K562 cells and K562 cells transfected to express either HLA-E*01:03 (HLA-E transfectant) or Mamu-E*02:04 (Mamu-E transfectant) were incubated with (**a**) the Mtb-derived RL9 peptide variant, or (**b**) the HIV Gag RL9 peptide indicated, washed to remove unbound peptide, then co-cultured with PBMC from (**a**) BCG-vaccinated, or (**b**) strain 68-1 RhCMV/HIVgag-vaccinated macaques in an ICS assay. Representative flow cyotmetric dot plots gated on CD3+CD8+ T cells (left columns) from one animal are shown, while CD8+ T cell response magnitudes from all three animals in each group are shown on the right.

Supplementary Figure 3:

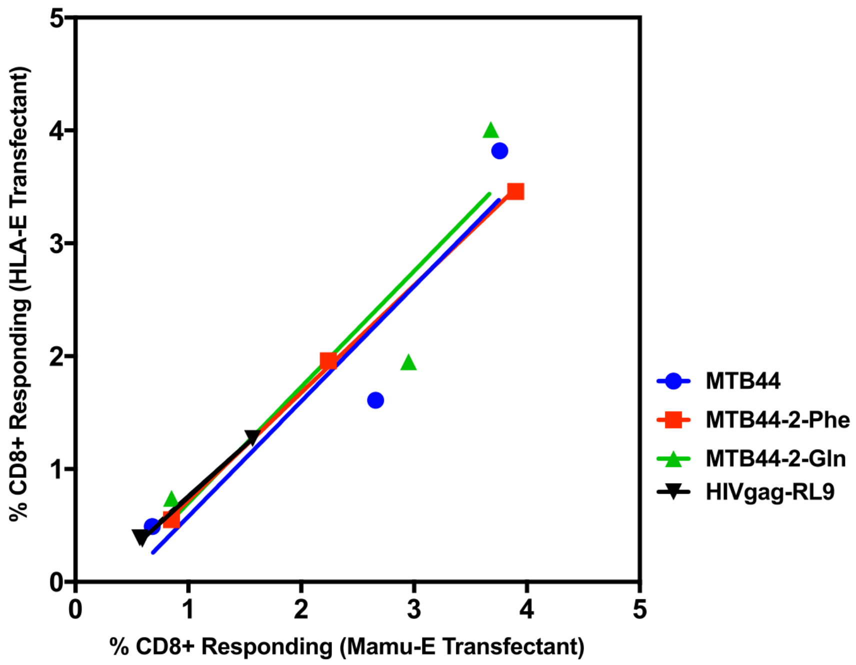

HLA-E and Mamu-E bind and present Mtb RL9 and HIV RL9 peptides equivalently to Mamu-E-restricted CD8+ T cells

The percentage of CD8+ T cells from BCG-vaccinated macaques (MTB RL9 variant peptides) or strain 68-1 RhCMV/HIVgag-vaccinated (HIVgag RL9peptide) macaques recognizing the peptide indicated in the context of presentation by HLA-E*01:03 (HLA-E transfectant) was plotted against recognition in the context of presentation by Mamu-E*02:04 (Mamu-E transfectant). No significant difference was observed.

Supplementary Table 1:

Data collection	HIA-E*01:03 in complex with peptide: Mtb44	Mtb44*P2-Gln	Mtb44*P2-Phe	Mtb44*P9-Phe	RL9HIV
PDB ID	6GH1	6GH4	6GGM	6GHN	6GL1
Crystallisation condition	2.4 M AS<br>0.1 M MES<br>pH 6	2.4 M AS<br>0.1 M HEPES<br>pH 7	2.0 M AS<br>0.1 M MES<br>pH 6.5<br>1mM Z_nSO_4	2.4 M AS<br>0.1 M HEPES<br>pH 7	2.4 M AS<br>0.1 M MES pH 6<br>0.05%<br>dichloromethane
Resolution Å	53.91 - 2.09<br>(2.13 - 2.09)	67.47 - 2.16<br>(2.20 - 2.16)	53.18 - 2.73<br>(2.78 - 2.73)	58.50 - 2.54<br>(2.59 - 2.54)	135.87 - 2.62<br>(2.66 - 2.62)
Space Group	P 1	P 1	P 1	P 1	C2
Cell dimensions	a = 48.35 Å<br>b = 76.31 Å<br>c = 111.02 Å<br>α = 99.26°<br>β = 99.50°<br>γ = 93.68°	a = 48.31<br>b = 76.41<br>c = 111.31<br>α = 99.39°<br>β = 99.83°<br>γ = 93.50°	a = 60.93<br>b = 66.04<br>c = 72.10<br>α = 102.30°<br>β = 101.22°<br>γ = 109.70°	a = 37.45<br>b = 64.68<br>c = 97.31<br>α = 107.03°<br>β = 93.89°<br>γ = 106.85°	a = 244.82<br>b = 48.22<br>c = 153.02<br>α = 90.0°<br>β = 117.38°<br>γ = 90.0°
Solvent content [%]<br>(molecules per AU)	46 (4)	44 (4)	57 (2)	51 (2)	45 (4)
Unique reflections	81176 (4505)	81277 (3904)	25095 (882)	26488 (1053)	48641 (2439)
Completeness [%]	89.1 (47.0)	98.0 (92.9)	95.9 (67.6)	97.7 (77.5)	100 (99.3)
R_{merge} [I]	0.09 (0.47)	0.18 (0.85)	0.23 (0.49)	0.21 (0.78)	0.31 (2.35)
I/sigma	2.8 (1.1)	4.3 (1.2)	6.3 (2.6)	5.2 (1.0)	5.2 (0.9)
Multiplicity	1.7 (1.5)	3.3 (2.6)	6.1 (2.4)	4.9 (2.3)	6.6 (6.7)
$CC_{1/2}$	0.99 (0.67)	0.98 (0.50)	0.96 (0.63)	0.98 (0.57)	0.98 (0.45)
**Refinement**					
No. of non-hydrogen atoms	13388	13602	6561	6486	12701
R_{factor} [%)]	0.191 (0.277)	0.202 (0.306)	0.180 (0.265)	0.218 (0.319)	0.223 (0.303)
R_{free} [%]	0.229 (0.336)	0.242 (0.368)	0.221 (0.308)	0.264 (0.389)	0.273 (0.316)
r.m.s.d. bonds [Å] §	0.002	0.003	0.005	0.006	0.003
r.m.s.d. angles [deg]	0.55	0.59	0.70	0.84	0.62
Ramachandran statistics					
Favoured [%]	98.21	98.3	98.5	98.52	98.17
Disallowed [%]	0	0	0	0	0

Crystallographic data collection and refinement statistics

AS: Ammonium sulphate. § r.m.s.d.: root mean square deviation from ideal geometry. Statistics for outer shell indicated in parentheses. AU: asymmetric unit. R_{free} equals the R-factor against 5% of the data removed prior to refinement.

Supplementary Table 2:

	**HLA-E*0103 - Mtb44**		
	**Peptide**	**Dist. Å**	**Heavy chain**
1	P1-Arg [N]	2.61	Tyr-171 [OH]
2	P1-Arg [O]	2.77	Tyr-159 [OH]
3	P2-Leu [N]	2.6	Glu-63 [OE2]
4	P2-Leu [N]	3.68	Tyr-7 [OH]
5	P7-Pro [O]	3.16	Asn-77 [ND2]
6	P9-Leu [N]	2.94	Asn-77 [OD1]
7	P9-Leu [O]	2.75	Tyr-84 [OH]
8	P9-Leu [O]	2.76	Ser-143 [OG]
9	P9-Leu [OXT]	3.18	Lys-146 [NZ]
	**HLA-E*0103 - RL9HIV**		
	**Peptide**	**Dist. Å**	**Heavy chain**
1	P1-Arg [N]	2.5	Tyr-171 [OH]
2	P1-Arg [N]	2.96	Tyr-7 [OH]
3	P1-Arg [O]	2.72	Tyr-159 [OH]
4	P2-Met [N]	2.82	Glu-63 [OE2]
5	P3-Tyr [OH]	3.46	Glu-152 [OE1]
6	P7-Ser [N]	3.28	Glu-152 [OE2]
7	P9-Leu [N]	2.88	Asn-77 [OD1]
8	P9-Leu [O]	2.73	Tyr-84 [OH]
9	P9-Leu [O]	2.84	Ser-143 [OG]
10	P9-Leu [OXT]	2.98	Lys-146 [NZ]
	**HLA-E*0103 - Mtb44*P2-Phe**		
	**Peptide**	**Dist. Å**	**Heavy chain**
1	P1-Arg [N]	3.2	Tyr-171 [OH]
2	P1-Arg [N]	2.65	Tyr-7 [OH]
3	P1-Arg [NE]	3.27	Glu-63 [OE1]
4	P1-Arg [O]	2.64	Tyr-159 [OH]
5	P2-Phe [N]	2.67	Glu-63 [OE1]
6	P2-Phe [N]	3.34	Tyr-7 [OH]
7	P7-Pro [O]	3.07	Asn-77 [ND2]
8	P9-Leu [N]	2.89	Asn-77 [OD1]
9	P9-Leu [O]	3.04	Tyr-84 [OH]
10	P9-Leu [O]	2.68	Ser-143 [OG]
11	P9-Leu [OXT]	3.5	Lys-146 [NZ]
	**HLA-E*0103 - Mtb44*P2-Gln**		
	**Peptide**	**Dist. Å**	**Heavy chain**
1	P1-Arg [N]	2.74	Tyr-171 [OH]
2	P1-Arg [NH1]	3.61	Thr-163 [OG1]
3	P1-Arg [O]	2.7	Tyr-159 [OH]
4	P2-Gln [N]	2.87	Glu-63 [OE2]
5	P2-Gln [NE2]	3.38	Thr-70 [OG1]
6	P7-Pro [O]	3.1	Asn-77 [ND2]
7	P9-Leu [N]	2.93	Asn-77 [OD1]
8	P9-Leu [O]	2.89	Tyr-84 [OH]
9	P9-Leu [O]	2.9	Ser-143 [OG]
10	P9-Leu [OXT]	2.64	Lys-146 [NZ]
	**HLA-E*0103 - Mtb44*P9-Phe**		
	**Peptide**	**Dist. Å**	**Heavy chain**
1	P1-Arg [O]	2.73	Tyr-159 [OH]
2	P2-Leu [N]	2.58	Glu-63 [OE2]
3	P7-Pro [O]	3.11	Asn-77 [ND2]
4	P9-Phe [N]	2.74	Asn-77 [OD1]
5	P9-Phe [O]	3.02	Tyr-84 [OH]
6	P9-Phe [O]	3.38	Ser-143 [OG]

Hydrogen bonding networks

Inter peptide-HLA-E heavy chain hydrogen bond formation with a donor/acceptor distance range of 2.5 to 3.5 Å.

Supplementary Table 3:

	B-pocket				D-pocket			C-pocket				E-pocket				F-pocket				
	9	24	45	67	97	99	159	70	73	74	116	133	147	152	156	81	95	118	123	124
**HLA-E**	**H**	**S**	**M**	**A**	**W**	**H**	**Y**	**T**	**I**	**F**	**F**	**W**	**S**	**E**	**Q**	**L**	**L**	**Y**	**Y**	**L**
Mamu-E*02:01:02	H	S	M	A	W	H	Y	T	**T**	F	F	W	S	E	Q	L	L	Y	Y	L
Mamu-E*02:02	H	S	M	A	W	H	Y	T	**T**	F	F	W	S	E	Q	L	L	Y	Y	L
Mamu-E*02:03	H	S	M	A	W	H	Y	T	**T**	F	F	W	S	E	Q	L	L	Y	Y	L
Mamu-E*02:04	H	S	M	A	W	H	Y	T	**T**	F	F	W	S	E	Q	L	L	Y	Y	L
Mamu-E*02:05	H	S	M	A	W	H	Y	T	**T**	F	F	W	S	E	Q	L	L	Y	Y	L
Mamu-E*02:07	H	S	M	A	W	H	Y	T	**T**	F	F	W	S	E	Q	L	L	Y	Y	L
Mamu-E*02:09	H	S	M	A	W	H	Y	T	**T**	F	F	W	S	E	Q	L	L	Y	Y	L
Mamu-E*02:10	H	S	M	A	W	H	Y	T	**T**	F	F	W	S	E	Q	L	L	Y	Y	L
Mamu-E*02:11	H	S	M	A	W	H	Y	T	**T**	F	F	W	S	E	Q	L	L	Y	Y	L
Mamu-E*0212:01	H	S	M	A	W	H	Y	T	**T**	F	F	W	S	E	Q	L	L	Y	Y	L
Mamu-E*0212:02	H	S	M	A	W	H	Y	T	**T**	F	F	W	S	E	Q	L	L	Y	Y	L
Mamu-E*02:13	H	S	M	A	W	H	Y	T	**T**	F	F	W	S	E	Q	L	L	Y	Y	L
Mamu-E*02:14	H	S	M	A	W	H	Y	T	**T**	F	F	W	S	E	Q	L	L	Y	Y	L
Mamu-E*02:15	H	S	M	A	W	H	Y	T	**T**	F	F	W	S	E	Q	L	L	Y	Y	L
Mamu-E*02:16	H	S	M	A	W	H	Y	T	**T**	F	F	W	S	E	Q	L	L	Y	Y	L
Mamu-E*02:17	H	S	M	A	W	H	Y	T	**T**	F	F	W	S	E	Q	L	L	Y	Y	L
Mamu-E*02:18	H	S	M	A	W	H	Y	T	**T**	F	F	W	S	E	Q	L	L	Y	Y	L
Mamu-E*02:19	H	S	M	A	W	H	Y	T	**T**	F	F	W	S	E	Q	L	L	Y	Y	L
Mamu-E*02:20	H	**F**	M	A	W	**Y**	Y	T	**T**	**L**	F	W	S	E	Q	L	L	Y	Y	L
Mamu-E*02:21	H	S	M	A	W	H	Y	T	**T**	F	F	W	S	E	Q	L	L	Y	Y	L
Mamu-E*02:22	H	S	M	A	W	H	Y	T	**T**	F	F	W	S	E	Q	L	L	Y	Y	L
Mamu-E*02:23	H	S	M	A	W	H	Y	T	**T**	F	F	W	S	E	Q	L	L	Y	Y	L

Alignment of key pocket-forming residues in HLA-E and Mamu-E allelic variants

HLA-E and Mamu-E allele sequences from IPD – MHC were aligned in ClustalW2 and key residues involved in primary and secondary pocket formation compared. Residues with contributions to multiple pockets were only included once.
